# Editorial: Diagnosis and management of allergy to chemotherapy and biologics

**DOI:** 10.3389/falgy.2023.1205345

**Published:** 2023-05-11

**Authors:** Ricardo Madrigal-Burgaleta, Mariana Castells

**Affiliations:** ^1^ Allergy & Severe Asthma Service, St Bartholomew's Hospital, Barts Health NHS Trust, London, United Kingdom; ^2^ Division of Allergy and Immunology, Department of Medicine, Brigham and Women's Hospital, Harvard Medical School, Boston, MA, United States

**Keywords:** drug allergy, drug desensitization, chemotherapy allergy, biologics, basophil activation test (BAT), drug provocation test, endophenotyes, personalized & precision medicine (PPM)

**Editorial on the Research Topic** Diagnosis and management of allergy to chemotherapy and biologics

The global burden of cancer is difficult to fathom, even knowing that the number of new cancer cases in the world in 2020 was 19,292,789, and the number of deaths was 9,958,133 (1). However, only when we put it into context do we realize that this means that someone is diagnosed with cancer every few minutes around the world (2).

Around 28% of these patients diagnosed with cancer need chemotherapy as part of their primary cancer treatment (2). Unfortunately, at least 5% of these patients will experience a hypersensitivity reaction to their chemotherapy (3).

On the one hand, recent data confirmed that patients who need to abandon chemotherapy because of hypersensitivity reactions have worse survival outcomes than those who can continue receiving treatment (4). On the other hand, we know that allergists can help allergic patients receive their first-choice therapies and maintain the same survival outcomes as their non-allergic counterparts by using drug desensitization (5, 6).

Allergists have the scope to help prolong countless lives worldwide.

Drug desensitization needs a complex setup and specific training but is cost-effective (6, 7). Regrettably, many allergy departments find this task daunting, resulting in great inequality in accessing this technique (7). Recent guidance from the World Allergy Organization (WAO) and the USA Updated Drug Allergy Practice Parameters has provided the practical framework to start helping these patients (7, 8). However, there are many grey areas, so the need for original research is dire (7).

We were delighted to edit this Research Topic for Frontiers in Allergy: *Diagnosis and Management of Allergy to Chemotherapy and Biologics*. The selected articles touched on many hot topics for which new evidence is essential to improve these patients' management further.

One of the outstanding achievements of the focus on drug hypersensitivity reactions to chemotherapy and biologics is that some discoveries have changed how we approach their classification (7).

The atypical symptoms triggered by these drugs and their differences in response to drug desensitization have spurred the description of new phenotypes and endophenotypes based on clinical history and biomarkers (9–11). Recent consensus documents described these phenotypes and endophenotypes as type I (mast cells/IgE or non-IgE-dependent), type II (cytotoxicity), type III (involving antigen-antibody immune complexes), type IV (mostly T-cell mediated), cytokine release reactions (CRR), and mixed reactions (type I and CRR) (7, 12).

However, some authors have observed that patients reacting to chemotherapy or biologics might experience an index reaction that fits with one endophenotype only to “convert” to a different endophenotype on subsequent exposure (9). It is still unclear whether patients are experiencing a newly discovered phenomenon or whether these are independent reactions to the same drug. However, Jimenez-Rodriguez et al. cast some light on the topic by describing their experience with drug desensitization in their cohort of 112 patients, where they identified 12 of these “converter” patients after reacting to taxanes, platins, and biologics.

Another problem allergists face is the logistic complexity linked to diagnostics; for example, skin testing can be expensive, intravenous drug challenge is risky, and both need a creative approach to hazardous drug management (7). Understandably, clinicians would appreciate access to validated *in vitro* techniques that could help them save on *in vivo* techniques. Specific IgEs are available for a few chemotherapy drugs, such as platins, and basophil activation testing (BAT) is a promising new tool (7, 13). Unsurprisingly, most efforts to explore the usefulness of BAT in reactions to chemotherapy have been placed on platins, as these are known to trigger mostly IgE-mediated reactions (13, 14). However, De Campos et al. described their practical experience in a prospective study where they found the optimal concentrations and cut-off points for BAT in taxane-reactive patients using CD63 and CD203c. In their selected population, the test showed a sensitivity of 53% and a specificity of 87%. The authors identified severe and IgE-mediated reactions as the most likely to have a positive result. In addition, the authors found two false positives in the control group. Their detailed methodology and results provide crucial information for future research in the field.

Except for a few remarkable exceptions, allergists are not usually present during the index reaction. However, it is useful to involve expert allergists even in the initial management of reactive patients (15). Not only can allergists ensure that all the aspects of the reaction are well-documented and valuable biomarkers are collected, but they can also help all these patients safely receive their treatments on that same day and fast-track the next steps of their allergy workup to avoid delays. To achieve this, Borrás Cuartero et al. designed an innovative approach where an allergist assesses infusion reactions in the oncology infusion centre and can organize empirical same-day desensitizations so that the treatment does not go to waste (15).

Type I desensitization mechanisms seem to involve mainly mast cells (14). However, preliminary data suggest that drug desensitization can affect drug-specific T-cell response due to the expansion of T-regulatory cells producing IL-10 and IL-35 (16). Still, there is some controversy about whether drug desensitization should be considered exclusively in immediate reactions mediated by mast cells (7). Nevertheless, even if some authors have successfully used drug desensitization in nonimmediate reactions, the consensus is that further research is needed (7). Vega et al. shared their experience using drug desensitization in 11 patients with nonimmediate reactions to different chemotherapeutic and biologic agents. Their data feature some exciting discoveries on the behaviour of nonimmediate reactions to these agents and challenge the traditional use of slow days/weeks-long desensitization protocols, which might not be appropriate for some chemotherapy agents.

Indeed, the concern for shorter desensitization protocols in patients reacting to chemotherapy and biologics comes mainly from trying to mimic the standard chemotherapy regime as much as possible. But also, efficiency, error control, or drug stability are other factors that have eventually spurred the development of single-bag desensitization protocols (13, 17). However, the consensus is that the effectiveness of this type of protocol needs to be ascertained in further studies (7). Opportunely, Kim et al. shared their extensive experience with over 1,000 single-bag desensitization procedures in 228 patients. Admittedly, this study has limitations, such as the lack of a complete allergy workup, which might incur selection bias. However, the very commendable efforts from this group in promoting single-bag desensitizations have been fruitful in motivating other groups to explore this technique and have turned it into a hot topic (7, 13).

Drug desensitization protocols are not cooking recipes that one can follow from a book. They can only be successful in the context of a highly complex setup led by expert allergists, in liaison with trained nurses and pharmacists, and indicated by specialists who are aware of their potential use (7, 18, 19). A recent WAO consensus document dedicated a whole section (including a practical, real-life example) to address how to redesign an allergy department to cope successfully with drug desensitization (7). One of the most recurrent concerns was handling hazardous drugs in allergy departments (7). Berges-Gimeno et al. tackle this extremely important topic. Their experience will be invaluable for any allergy department willing to help patients who react to chemotherapy and biologics.

Last but not least, we thought the strong presence of female authors in these articles was worth mentioning. Despite disadvantages for women in the rules of engagement, it seems that the allergy speciality is starting to embrace the challenge and add female value (20, 21).

Thanks to these featured articles, we are exploring new endophenotypes of reactions, how to better diagnose patients, how to perfect the management of their reactions from the onset, how to better protect patients and staff from hazards, and how to streamline processes. In conclusion, all these articles have helped emphasize the crucial importance of the role of the allergist in dealing with patients who experience reactions to chemotherapy and biologics.
